# Eating habits of children and adolescents during the COVID-19 era: A systematic review

**DOI:** 10.3389/fnut.2022.1004953

**Published:** 2022-10-18

**Authors:** Farzad Pourghazi, Maysa Eslami, Amir Ehsani, Hanieh-Sadat Ejtahed, Mostafa Qorbani

**Affiliations:** ^1^Endocrinology and Metabolism Research Center, Endocrinology and Metabolism Clinical Sciences Institute, Tehran University of Medical Sciences, Tehran, Iran; ^2^Department of Pediatrics, Alborz University of Medical Sciences, Karaj, Iran; ^3^Obesity and Eating Habits Research Center, Endocrinology and Metabolism Clinical Sciences Institute, Tehran University of Medical Sciences, Tehran, Iran; ^4^Non-Communicable Diseases Research Center, Alborz University of Medical Sciences, Karaj, Iran

**Keywords:** COVID-19, children, adolescence, eating habits, pandemic

## Abstract

The COVID19 pandemic has affected all aspects of people's lives. Eating habit plays a crucial role in children and adolescents' physical and mental development and the impacts might last until adulthood. This systematic review aimed to summarize a comprehensive and updated overview of eating habits changes due to COVID19 confinements among children and adolescents. A systematic literature search was performed in three databases for all the English studies published from the start of the confinements until April 2022. Two researchers screened articles independently and included observational studies which evaluated children's and adolescents' eating habits before and during confinements. The quality of the included studies was assessed by Newcastle-Ottawa Quality Assessment checklists for cross-sectional and cohort studies. Among 2,436 studies, 39 final full-text articles were included. The total participants of this systematic review consist of 157,900 children and adolescents. Seven categories were identified: daily eating patterns, junk food, beverage, fruits and vegetables, milk and dairy, protein-rich foods, and legumes and cereals. In summary, most of the included studies reported a significant increase in consumption of home-cooked meals, amount of food, snack, french fries, sweets, fruits, vegetables, legumes, bread, and bakery products. On the other hand, studies demonstrated significantly lower intake of fast food and soft drink. The studies reported controversial results about breakfast consumption, sugar-added drinks, caffeinated drinks, milk and dairy products, protein-rich foods (including meat, fish, egg and chicken, and poultry), rice, and cereal. Changes in children's and adolescents' eating habits during the COVID-19 era were both positive and negative, for example, a decrease in fast food, fruit, and vegetable consumption vs. an increase in snacking and sweet consumption. Both changes have significant short-term and long-term impacts on population health. This study could provide us with insight into the changes in eating habits in children and adolescents in the COVID-19 era which we can use to limit the negative consequences on health.

## Introduction

Due to the spread of the coronavirus disease, the imposed limitations to control the disease have led to many changes in millions of people's lives worldwide from the late 2019 until today (1). In addition to social distancing, lifestyle habits have changed during the COVID-19 era (2). Physical inactivity (3), increased screen time (4), psychological distress (5), and disruption of sleep patterns (6) have been reported during repeated confinements. As a result, these abrupt changes affected health behaviors, including eating habits, across all age groups (7). School closure, online teaching, and outdoor activity restriction were other dimensions that significantly impacted children and adolescents' lifestyles and eating behaviors (8).

Eating habit is “the habitual decisions of individuals or a group of people regarding what food they eat” (9) which was affected by COVID-19. Diet and eating habits play an important roles in children and adolescents' physical and mental development (10, 11). Any unhealthy eating habits in the growth period of life can lead to irreversible consequences such as the increased risk of obesity, non-communicable diseases, and decreased immune system function (12, 13).

Although some review studies showed that the pattern of consuming snacks, home-cooked food, junk foods, and fruits and vegetables increased significantly, on the other hand, some other studies showed controversial findings (1, 14–16).

Therefore, this systematic review was designed to assess the effect of the COVID-19 era on dietary habits in children and adolescents.

## Methods and materials

All research steps were conducted according to the preferred reporting items for systematic reviews and meta-analyses (PRISMA) statements in this systematic review (17).

### Search strategy

A systematic literature search was performed to identify the impact of the COVID-19 epidemic on eating behaviors in children and adolescents. The electronic search was conducted in three international databases (Pubmed, Scopus, and Web of Science) for all the English studies published from the start of the confinements until April 2022. Moreover, Google scholar was also searched and reference checking was done. The following search terms were used in this systematic search, with the minimum restriction possible: (“eating habits”[Title/Abstract] OR “dietary intake”[Title/Abstract] OR “dietary pattern”[Title/Abstract] OR “food choices”[Title/Abstract] OR “diet quality”[Title/Abstract] OR “eating behaviors”[Title/Abstract] OR “food preferences^*^”[Title/Abstract]) AND (“covid-19”[Title/Abstract] OR “SARS-CoV-2”[Title/Abstract] OR “coronavirus”[Title/Abstract] OR “covid19”[Title/Abstract]).

### Study selection

All references were initially imported to End Note version 9.3.3, and duplicates were detected and removed. Two researchers FP and ME screened titles and abstracts in line with inclusion and exclusion criteria. The remaining studies that seemed potentially relevant were reviewed in their full text. Any conflicts at any stage of screening were discussed and resolved by two authors and senior authors.

### Inclusion criteria

- Papers investigated any changes (qualitative or quantitative) in eating behavior, including consumption of foods and beverages or following a specific diet.
- Exposure was lockdowns resulting from COVID-19, and studies compared eating habits with before lockdowns
- The population of interest was children and adolescents
- Observational studies, including cross-sectional, case-control, prospective, or retrospective cohort studies
- English-language studies.

### Exclusion criteria

- Eating habit changes were not compared before and during lockdowns
- Papers that evaluated eating habits only before OR during confinements
- Participants were not children or adolescents
- Clinical trials, reviews, books, and conferences.

### Data extraction

The following items were extracted from eligible studies:

- General characteristics of the studies include the first author's name, year, and country.
- Methodological characteristics of the studies include study design, the population of the study, sample size, assessment tool, and target habit.
- Outcomes of the studies include changes in daily eating patterns (including the number of meals, eating breakfast, amount of foods and home-cooked foods) and different groups of foods and beverages and their subgroups, including Junk Foods (snack, fast food, French fries and chips, processed food and sweets), beverages (soft drink, sugar added drink, caffeinated drink), fruits & Vegetables, Milk and Dairy, Protein Sources (meat, egg, fish, chicken), Legumes & Cereals (rice, soybean, cereal, bread, and bakery).

### Quality assessment

The quality of the included studies was assessed by Newcastle-Ottawa Quality Assessment Form for cross-sectional and cohort studies (18, 19). FP and ME independently appraised the studies' quality using Newcastle-Ottawa quality assessment checklists. The Newcastle-Ottawa scale evaluates the methodological quality of the studies in seven items for cross-sectional and eight items for cohort studies within three categories: (1) Selection of participants (maximum 4 scores), (2) Comparability of subjects (maximum 2 scores), (3) Assessment of outcome (maximum 3 scores). We classified the quality of each study as follows: for cross-sectional studies 9 and 10 points as “very good,” 7 and 8 points as “good,” 5 and 6 as “satisfactory,” and 0 to 4 as “unsatisfactory.” For cohort studies, if a study gets 3 or 4 points in the selection part AND 1 or 2 points in the comparability part, AND 2 or 3 points in the outcome part, it is considered “good quality.” If a study gets 2 scores in the selection part AND 1 or 2 scores in the comparability part AND 2 or 3 points in the outcome part, it is considered “fair quality,” and if a study scored 0 or 1 in the selection part OR 0 stars in comparability part OR 0 or 1 stars in outcome part, it is considered as “poor quality.”

## Results

### Search results

**Figure 1:**
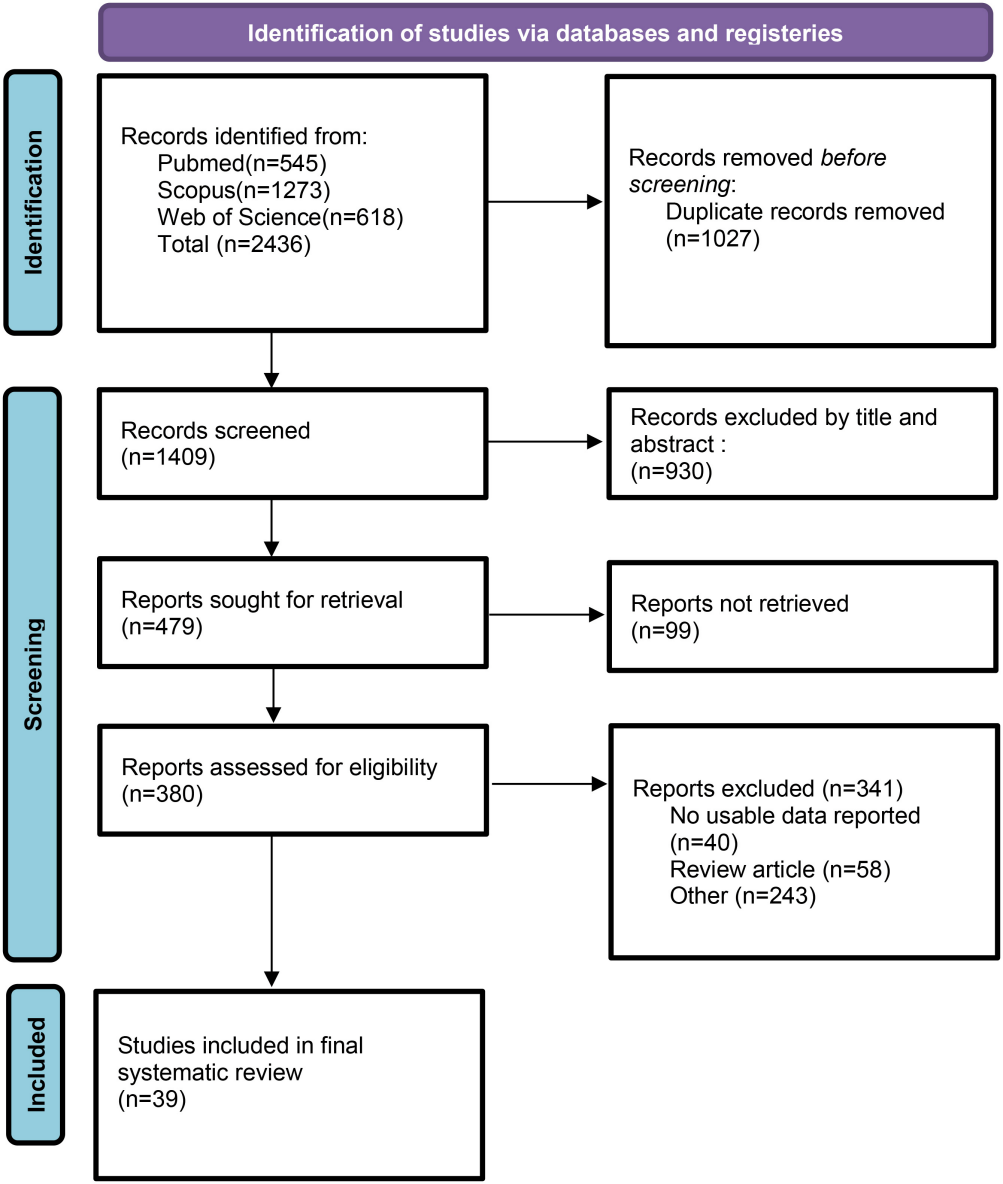
Flow chart for study identification and selection. Based on PRISMA 2020.

The screening steps are shown in the PRISMA flow diagram (Figure 1). The initial electronic search of three databases retrieved 2,436 articles (Pubmed = 545, Scopus = 1,273, Web of science = 618). One thousand and twenty seven articles were detected as duplicates and removed. After reviewing titles and abstracts, 930 articles were excluded. Sixty four articles did not have an observational design, and 35 articles were excluded because of being book chapters and conferences. After that, 380 papers were reviewed for full text. Papers that did not specifically compare eating habits before and during COVID-19 lockdowns were deprived. Finally, 39 articles met the inclusion criteria for this systematic review (2, 5, 8, 20–55).

### Description of studies

Characteristics of the included studies are presented in Table 1. Studies were conducted on all five continents. Nearly half of the studies (48%) were from Europe (Spain = 5, Poland = 4, Italy = 3, France = 2, Germany = 1, Netherland = 1, Greece = 1, Croatia = 1). Nine studies were conducted in America (United states = 5, Brazil = 3, Canada = 1), eight from Asia (Palestine = 2, China = 2, Saudi Arabia = 1, Japan = 1, South Korea = 1, Jordan = 1), and two from Africa (Egypt = 2), and one from Australia. One international study was performed in five countries (Spain, Italy, Brazil, Colombia, and Chile). Among 39 articles, 35 were cross-sectional studies, three cohorts, and one quasi-experimental. The total participants of this systematic review consist of 157,900 children and adolescents. Questionnaires were mostly employed as the assessment tool. Due to pandemic confinements and limitations, all studies had online or telephone interviews, except for three studies for which the interviews were held face-to-face, with health protocols being observed, following the cancelation of COVID-19 confinements. About 12 studies had prepared validated questionnaires that improved the study's quality.

**Table 1:** General characteristics of included studies.

**References**	**Country**	**Study design**	**Study population**	**Sample size**	**Assessment tool**	**Target habit**	**Quality assesment score**
Adams (21)	United States	Prospective Cohort	Parents (*N =* 433) ≥18 years of age, living in the United States, and with a child 5–18 years of age completed online surveys May and September 2020 during COVID-19	*N =* 433 Boys: 214 Girls: 219	Study Specific questionarre	Parent reported food security, home food environement, parent feeding excercises before and during Covid-19	5
Adams (20)	United States	Cross-Sectional	Parents living in the United States, who were 18 years of age or older, and had at least 1 child between 5–18 years of age were eligible	*N =* 584 Boys: 280 Girls: 304	Study Specific questionarre	Parent reported home food environment and parent feeding practices by different food security status	2
Aguilar (22)	Spain	Cross-Sectional	14–18 year-old students from a secondary school in Central Catalonia	*N =* 303 Boys: 91 Girls: 212	Validated Study Specific questionarre (DeskCohort Survey)	Frequency of consumption and eating behavior socioeconomic and health related variables also evaluated	2
Alamrawy (24)	Egypt	Cross-Sectional	Egyptian participants aged 14–24 years	*N =* 447 Boys: 133 Girls: 314	Study Specific questionarre	Dietary changes during the COVID-19 pandemic according psychiatric morbidities	1
Al Hourani (23)	Jordan	Cross-Sectional	Children and adolescents aged 6–17 y	*N =* 477 Boys: 231 Girls: 245	Validated Study Specific questionarre FFQ	Food intake change before and during Covid-19 evaluated	4
Allabadi (25)	Palestine	Cross-Sectional	Adolescents 10–19 year age	*N =* 600 Boys: 300 Girls: 300	Study specific questionarre	dietary habits, physical activity, screen time, sleeping patterns, sources of stress according to sex	2
Androutsos (26)	Greece	Cross-Sectional	Parents who have one child aged 2–18 years	*N =* 397 Boys: NR Girls: NR	Study Specific questionarre	Dietary habits and life styles before and during Covid-19 and correlation with weight change	1
Beck (27)	United States	Cross-sectional	Parents of children ages 4–12 with body mass index ≥85th percentile	*N =* 145 Boys: 65 Girls: 80	Study Specific questionarre	child health behaviors before and after the start of the pandemic and family food insecurity before and after the start of the pandemic	1
Burkart (8)	United States	Quasi-Experimental	Children aged 7–12 y	*N =* 74 Boys: 39 Girls: 35	Validated questionnaire (Beverage and Snack questionarre)	Parents reported their child's screen time and dietary intake before and during Covid-19	5
Cipolla (5)	Italy	Cross-sectional	Children and adolescents age between 8 and 18 with overweight and obesity	*N =* 64 Boys: 26 Girls: 38	Study Specific questionarre	Eating habits before and during covid-19 and relation with obesity and psychological factors	1
Dragun (28)	Croatia	Cross-sectional	Secondary school students,domestic medical students,international medical students	*N =* 1,326 Boys: 477 Girls: 842 Pre lockdown and 531 Boys: 147 Girls: 384 during lockdown	Validated Study Specific questionarre MDSS	Snacking while watching TV Breakfast frequency and psychological well-being pre and during Covid-19	1
Ferrante (29)	United States	Cross-sectional	Parents with 4–8 y old children	*N =* 1,000 Boys: 450 Girls: 550	Study Specific questionarre	Family food acquisition and eating behaviors (e.g., cooking, restaurant use)	2
Giannini (30)	Brazil	Cross-sectional	Adolescents aged between 12 and 18 y	*N =* 208 Boys: 91 Girls: 119	Study Specific questionarre	Eating behavior change, Breakfast and relation with psychological morbidities	1
Glabska (31)	Poland	Cross-sectional	Secondary school students in a national sample of Polish adolescents	*N =* 2,448 Boys: 896 Girls: 1,552	Study Specific questionarre	Food choice questionnaire before and during Covid-19	5
Hanabazaza (32)	Saudi Arabia	Cross-sectional	Children aged 6–15 y	*N =* 280 Boys: 143 Girls: 137	Study Specific questionarre	Eating habits, physical activity, and sedentary behavior	3
Hashem (33)	Egypt	Cross-sectional	Parents of children and adolescents after two whole months of lockdown and school closure in Egyp	*N =* 765 Boys: 408 Girls: 357	Study Specific questionarre	Dietary pattern, eating behavior, and physical activity	2
Horikawa (34)	Japan	Cross-sectional	10–14 year old children	*N =* 1,111 Boys: 545 Girls: 566	Study specific questionarre	The relationship between household income and the quality of meals in Japanese school children before, during, and after the state of emergency.	6
Jia (35)	China	Cross-sectional	Participants in at three educational level aged 15–28 years	*N =* 10,082 Boys: 2,852 Girls: 7,230	Validated study specific questionarre (COINLICS)	Consumption pattern of 12 food group and beverages across educational level, sexes and before and during Covid-19	5
Kim (36)	South Korea	Cross-sectional	The 12–18-year-old population	*N =* 105,600 Boys: 54,809 Girls: 50,791	Validated Study Specific questionarre (KYRBWS Survey)	Dietary habits and exercise pattern before and during Covid-19	8
Kolota (37)	Poland	Cross-sectional	Primary school students adolescents aged 10–16 years	*N =* 1,334 Boys: 623 Girls: 711	Validated Study specific questionarre	The study assessed the diet and physical activity of the participants	6
Lopez Bueno (38)	Spain	Cross-sectional	Parents of children and adolescents aged between 3 and 16 y	*N =* 860 Boys: 437 Girls=423	Study specific questionarre	Physical activity, screen exposure, sleep time, and fruit and vegetable consumption before and during the Covid-19 confinement.	3
Luszczki (39)	Poland	Cross-sectional	Children and adolescents aged 6–15 y	*N =* 1,016 Boys: 495 Girls: 521	FFQ-6 validated questionarre	Eating behaviors, level of physical activity (PA), hours of sleep, and screen time among Polish children and adolescents before and during the COVID-19	8
Malta (40)	Brazil	Cross-sectional	Brazilian adolescents aged 12–17	*N =* 9,470 Boys: 4,712 Girls: 4,758	Study specific questionarre	Changes that occurred in the lives of Brazilian adolescents during the isolation period	4
Maximova (41)	Canada	Cross-sectional	Grade 4–6 students (age 9–12 years) from 20 schools socioeconomically disadvantaged	*N =* 1,095 Boys: 538 Girls: 557	Study specific questionarre (In person)	lifestyle behaviors, mental health and wellbeing during the lockdown.	6
Mazzolani (42)	Brazil	Cross-Sectional	Adolescents with multiple chronic conditions aged between 10 and 18 years and healthy controls	*N =* 348 Boys: 217 Girls: 131	questionnaire	Eating habits and sedentary behavior	2
Medrano (43)	Spain	Cross-sectional	Schoolers aged 8–16 years	*N =* 113 Boys: 55 Girls: 58	KIDMED online questionnaire	Physical activity, screen time, sleep time, adherence to the Mediterranean diet and sociodemographic information were longitudinally assessed before and during lockdown	2
Munasinghe (44)	Australia	Prospective Cohort Study	A cohort of young people aged 13–19 years were prospectively followed for 22 weeks	*N =* 582 Boys: 102 Girls: 465	Validated questionarre	Daily, weekly, and monthly trajectories of diet, physical activity, sedentary behavior, well-being, and psychological distress were	7
Perrar (45)	Germany	Prospective Cohort	Children and adolescents 3–18 years old in Germany	*N =* 108 Boys: 63 Girls: 45	Study specific questionarre	Nutrients and food intake repeated 3-day weighed dietary records	7
Philippe (46)	France	Cross-Sectional	Parents of 498 children aged 3–12 years	*N =* 498 Boys: 238 Girls: 260	Validated Study specific questionarre (CEDQ,CEBQ, HomeSTEAD)	Changes in child eating behaviors, parental feeding practices, and parental motivations when buying food during the lockdown, compared to before the lockdown.	7
Philippe (47)	France	Cross-Sectional	Parents of 498 children aged 3–12 years	*N =* 498 Boys: 238 Girls: 260	Study specific questionarre	Parents (*N =* 498, 72% mothers) of children aged 3-12 years described which food-related changes they (1) perceived as positive during the lockdown, (2) perceived as negative, and (3) would like to maintain after the lockdown.	2
Pietrobelli (48)	Italy	Cross-Sectional	The sample included 41 children and adolescents with obesity participating in a longitudinal observational study	*N =* 41 Boys: 22 Girls: 19	Study specific questionarre (Interview in person and telephone)	Lifestyle information including diet, activity, and sleep behaviors was collected at baseline and 3 weeks into the national lockdown	1
Pujia (49)	Italy	Cross-Sectional	The parents of children (5–9 years) and adolescents (10–14 years)	*N =* 439 Boys: 246 Girls: 193	CREA validated questionnare	demographic and anthropometric data and dietary habit changes during the COVID-19 lockdown	4
Radwan (2)	Palestine	Cross-Sectional	Primary and secondary school students aged 6–18 year old	*N =* 2,398 Boys: 487 Girls: 1,911	Validated Study specific Questionarre	socio-demographic, eating habits as well as quality and quantity of food intake	2
Ramos Alvarez (50)	Spain	Cross-Sectional	Children aged 11–12	*N =* 50 Boys: 33 Girls: 17	Validated instrument for assess dietary intake(Alpha-Fitness Battery)	Dietary intake, habits and practices	5
Ruiz Rosso (51)	Spain Italy Brazil Colombia Chile	Cross-Sectional	Adolescents aged 10–19 years,	*N =* 820 Boys: 319 Girls: 501	Validated questionarre (PeNSE)	More than 30 questions about dietary habits during COVID-19 confinement and the previous period	6
Vall Roque (52)	Spain	Cross-Sectional	Aged 14–35 years old	*N =* 2,837 Boys: 128 Girls: 2,719	Study specific questionarre	Depression, anxiety, stress, self-esteem, and disordered eating measures	6
Welling (53)	Netherland	Cross-Sectional	Parents of children 0–18 year old with severe obesity	*N =* 83 Boys: 40 Girls=43	Validated questionnaire	Effects of the lockdown measures on the children's lifestyle behaviors	3
Yu (54)	China	Cross-Sectional	Youth participants under three educational attachments (i.e., high school, college or graduate) in China	*N =* 10,082 Boys: 2,852 Girls: 7,230	Study specific questionarre	The sociodemographic information and routine dietary patterns before and after lockdown of participants were investigated	6
Zachurzok (55)	Poland	Cross-Sectional	Participants with a mean age of 12.8 ± 2.6 years admitted to three pediatric endocrinology clinics	*N =* 177 Boys: 81 Girls: 96	Study specific questionarre	Eating habits, physical activity, screen time, and sleep before and during the lockdown	1

N, Number; NR, Not Reported.

Concerning the quality assessment of the studies, out of 39 studies, 15 were considered good or satisfactory quality, and 24 had low quality (Supplementary Table S1).

**Figure 2:**
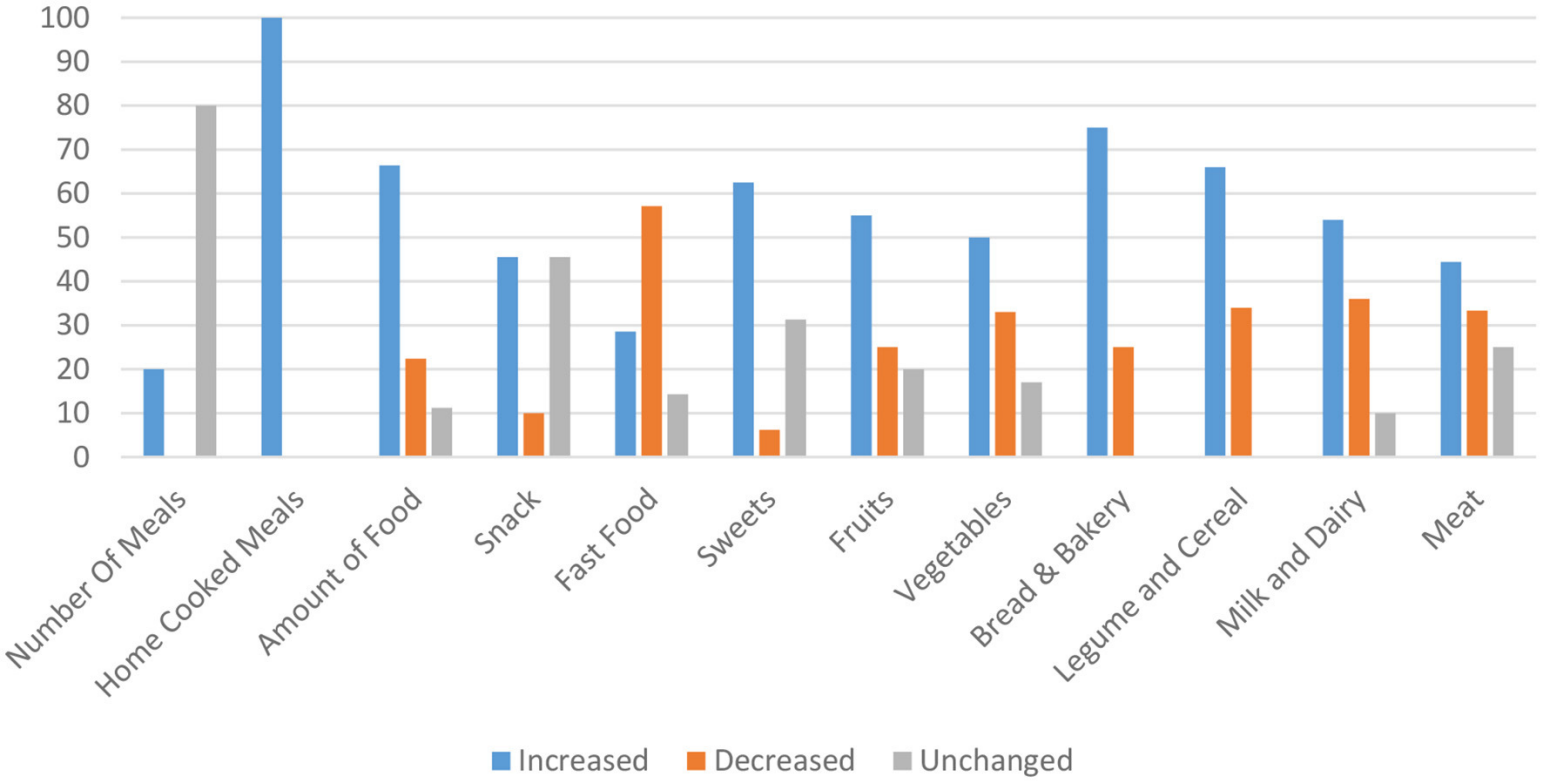
Summary of eating habits changes according to percentage of studies.

As shown in Table 2, data were categorized into seven groups, including daily eating patterns, junk food, beverages, fruits and vegetables, dairy and milk, protein-rich foods, legumes, and cereals. To have a simple outlook and rapid overview, changes in each item were illustrated with arrows. Generally, sweets, fruits, vegetables, and fast food were the most evaluated items by the studies. Any kind of dessert, chocolates, and cake was considered in the sweets category in this review. In summary, most of the included studies reported a significant increase in consumption of home-cooked meals, Amount of food, snack, french fries, sweets, fruits, vegetables, legumes, bread, and bakery products. On the other hand, most studies demonstrated significantly lower intake of fast food and soft drink. The studies reported controversial results about the consumption of breakfast, sugar-added drinks, caffeinated drinks, Milk and dairy products, protein-rich foods (including meat, fish, egg and chicken, and poultry), rice, and cereal (Figure 2).

**Table 2:** Observed changes in eating habits in seven categories due to COVID-19 lockdowns in the 39 included studies.

**References**	**Daily eating patterns**				**Junk foods**					**Beverages**			**Fruits and vegetables**		**Milk and dairy**	**Protein rich foods**				**Legumes and cereals**				
**Food type**	**Number of meals**	**Breakfast**	**Amount of food**	**Home cooked**	**Snack**	**Fast food**	**French fries and chips**	**Processed food**	**Sweets**	**Soft drink**	**Sugar added**	**Caffeinated**	**Vegetables**	**Fruit**	**Milk and dairy**	**Meat**	**egg**	**Fish**	**Chicken (Poultry)**	**Legumes**	**Rice**	**Soy bean**	**Cereal**	**Bread and bakery**
Adams (21)			—		—			—	—															
Adams (20)			↑	↑	**—**	↓		↑	—															
Aguilar (22)	—		↑		↑			↓	↓	↓			↑	↑	↑	↓	↑	↓		↓			↑	
Alamrawy (24)	—									—		—												
Al Hourani (23)						6–12 y: — 13–17y: ↑	6–12 y: ↑ 13–17y: ↑		6–12 y: ↑ 13–17y: ↑	6–12 y: ↑ 13–17y: ↑		6–12 y: — 13–17y: ↑	6–12 y: ↑ 13–17y: ↑	6–12 y: ↑ 13–17y: ↑	6–12 y: ↑ 13–17y: ↑	6–12 y: ↑ 13–17y:	6–12 y: ↑ 13–17y: ↑	6–12 y: — 13–17y: —	6–12y: ↑ 13–17y: —		6–12 y: ↑ 13–17y: ↑		6–12 y: — 13–17y: ↑	6–12 y: ↑ 13–17y: ↑
Allabadi (25)			↑			↑		—	↑		↑		↑	↑	—									
Androutsos (26)		↑			↑	↓			↑	—			↑	↑	↑	—		—	—					
Beck (27)									—		—		—	—										
Burkart (8)*																								
Cipolla (5)						↑			↑															↑
Dragun (28)		—			—				↑					↑	↓			↑		↑			↓	
Ferrante (29)				↑		↓																		
Giannini (30)			↑																					
Glabska (31)*																								
Hanabazaza (32)		↓				↓			—	—	—		—	—	↓									
Hashem (33)			↑		↑				↑															
Horikawa (34)													↓	↓	↓	↓	↓	↓						
Jia (35)											↓		↓	↓	↓	↓			↓		↓	↓	↑	
Kim (36)		↑				↓				↓	↓			↓										
Kolota (37)						—	—			—			↑	↑										
Lopez Bueno (38)													↓	↓										
Luszczki (39)					↓	↓		↓	↑	↓			—	—	↑	↑		↑	↑	↓				↓
Maximova (41)	—				↑																			
Mazzolani (42)				↑				↓																
Medrano (43)		↓																		↑				
Malta (40)							12–15y: ↓ 16–17y: ↑		↑				↑	—										
Munasinghe (44)						↓							—	—										
Perrar (45)			↓																					
Philippe (46)					—		↑		↑	↑				↑	↑									
Philippe (47)*																								
Pietrobelli (48)	↑						↑				↑		—	↑		↑								
Pujia (49)					↑			↑		↓			↑	↑		↑				↑				↑
Radwan (2)			↓	↑		↓																		
Ramos Alvarez (50)*																								
Ruiz Rosso (51)						↓			↑		—		↑	↑		—				↑				
Vall Roque (52)*																								
Welling (53)			↑																					
Yu (54)											↓	↑	↑	↑	↑		↑	↑			↓		↑	
Zachurzok (55)	—				—	—			—	—		—	—											

*Findings of these studies were qualitative and were not mentioned in the table.

A few studies reported that eating habit changes qualitatively. Medrano et al. evaluated adherence to the Mediterranean diet using the Mediterranean Diet Quality Index for children and teenagers (KIDMED) questionnaire (43). They observed that the KIDMED score increased among participants during confinements (43). In another study, dietary intake increased, and the pattern changed to unhealthy foods (unhealthy:+1.2 foods, 95% CI = 1.0, 1.5) (8). A study in Spain demonstrated that eating habits for 44.6% of women aged 14–24 years deteriorated since the lockdowns started, while 24.8% reported no change and 30.5% reported their eating habits got better (52).

## Discussion

To our knowledge, this is the first comprehensive systematic review that evaluated the impacts of the confinements during the COVID-19 era on children's and adolescents' eating habits.

As mentioned, we divided findings into categories to simplify assessing the results and understanding how children's and adolescents eating habits changed. We could see different results among studies, even those from the same country. Many parameters influence the obtained results.

### Daily eating patterns

The findings of the studies, in this case, were equivocal. All of the studies reported no change in the number of meals per day except one study, which was a study in Italy that included 41 children and adolescents. The information in this study was collected before COVID-19 started and just 3 weeks after the mandatory national quarantine started (48). An important factor influencing the outcome is the time the study was done. Included articles were conducted at different quarantine periods, for example, beginning, middle, or after the confinement. A youth's psychiatric condition, such as depression, anxiety, or insomnia, is significantly associated with her/his eating habits, including the number of meals, increased consumption of caffeinated or energy drinks, etc., (24). As a result of stress, boredom, and increased screen time led to irregularity in meal distribution (22).

There are controversies among studies' reports about breakfast consumption change during confinements. Two studies have reported a decrease in breakfast consumption during confinement compared with pre-pandemic (32, 43). On the other hand, another two studies indicated an increase (26, 36). One study did not find a significant difference in the frequency of breakfast consumption in their participants (28). Sleep patterns have changed during the COVID-19 era, and inadequate sleep is related to unhealthy diets such as skipping breakfast (37). Skipping breakfast is related to a higher risk of overweight and obesity in children and adolescents (62). On the other hand, due to school closure, children had more time to have regular meals. It is worth considering that the quality of the four studies was unsatisfactory except for Kim et al.'s study, a survey with 105,600 participants conducted in South Korea (36).

Confinements and stay-home orders led to positive changes in daily lifestyle, one of which was the increased consumption of home-cooked meals (2, 20, 29, 42). Closure of shops, malls, and restaurants or limited open hours have made respondents shift to home-cooked meals (2). Consuming more home-cooked food provides spending more time with family by participating in cooking, eating together, and communicating (20, 42). Parents are generally responsible for providing food for their children, so they should be educated about preparing healthy foods and building healthy eating habits (20, 42).

The amount of food is one of the important factors that identifies the household food environment and food security (20, 21). Most of the included studies have indicated an increase in the amount of food children and adolescents consumed during COVID-19 confinements compared with pre-pandemic (21, 22, 25, 30, 33, 53). In the families who became food insecure or stayed insecure, the amount of consumed foods has decreased (21). Economic crisis and loss of the source of income during a pandemic might limit access to food in disadvantaged families (2).

In accordance with our findings, in a systematic review by Mignogna et al., conducted among all age groups, an increase in food intake and the number of daily meals were observed (56).

### Junk foods

We considered the most frequently evaluated junk food items in the included studies: snacks, fast foods, French fries or chips, processed food, and sweets.

It was hypothesized that stress, boredom, and long screen time exposure during confinements are associated with frequent snacking. Among the included studies in this review, five studies showed increased snacking. TV watching, laptop, and mobile screen time were significantly associated with frequent snacking between meals and at night (21). Other five articles reported no change in consumption of snacks (20, 21, 28, 46, 55). In a cohort study by Adams et al., unhealthy snack intake increased in May 2020 compared to before COVID-19. A few months later, in September 2020, participants' calorie-dense snack intake decreased. We can see that families and children adapted to confinement conditions over time (21).

Most studies indicated that fast food consumption decreased (2, 20, 26, 29, 32, 36, 39, 44, 51). As mentioned above, in the home-cooked category, fast food consumption decreased due to the closure and limited open hours of restaurants, malls, and cafes or the fear of becoming infected by the person delivering the food (2). In addition, parents and children tended to eat more healthy and homemade foods to enhance their health and immunity. The characteristic of the participants could be an important factor that has an impact on the obtained result. Clearly, children's eating habits differ from adolescents', or subjects with underlying diseases might have a specific diet compared with a healthy one (46). One study showed a significant increase in pizza consumption in those aged 13–17 compared with those aged 6–12 (23). Another study reported increased consumption of pizza, bread, and pasta to fill the time in children and adolescents who are overweight or suffering from obesity, and this study showed that the lockdowns worsened participants' eating habits (5).

“Convenience food” was considered processed food in this study. Three studies showed a decrease (22, 39, 42), and two showed no change in consumption of processed food (21, 25). The authors noted that the consumption of fast foods was reduced after “stay-home” orders. The decrease in processed and convenience food correlates with reduced fast food intake (22, 39). However, two studies reported an increase in processed food intake. Due to supply chain issues for healthy animal proteins, vegetables, and fruits during the pandemic, the costs increased. Therefore, Eating preferences were changed toward cheap and high-calorie foods like processed foods (24).

It is obvious that having lots of French fries is an unhealthy eating habit. Almost all the included articles pointed to increased consumption of French fries or potato chips (23, 46, 48).

In line with previous studies in children and adolescents or adults (56), most of the included studies (ten) indicated an increase in comfort unhealthy food consumption like sweets, desserts, pastries and cakes, candy, and chocolates in participants during confinements (5, 23, 25, 26, 28, 33, 39, 40, 46, 51). Eating is one of the coping mechanisms to reduce the intensity of negative stressors related to pandemics (63). Foods rich in sugar and fat are preferred as pleasant and instant rewards to distract a person from experiencing negative emotions (64). Only one study in Spain showed that intake of sweets and pastries decreased, especially in boys, and explained the lower social interactions during confinements could partly describe this change (22).

### Beverages

Generally, studies have shown discordant results in the consumption of different types of beverages and their changes during confinements. Most of the studies indicated no change in consumption of soft drinks, sugar-added drinks, and caffeinated drinks (24, 26, 27, 32, 37, 51, 55). Four studies reported a lower amount of soft drink consumption during COVID-19 than before confinement (22, 36, 39, 49). During Confinement eating out decreased, which could explain the lower consumption of soft drinks. By contrast, two studies showed a higher intake of soft drinks (23, 46). These controversies can be explained due to geographical differences and the time of studies. For example, one of the studies reported a higher intake of soft drinks conducted during the holy month of Ramadan in Jordanian adolescents (23).

Coffee is a good source of energy. Adolescents especially use coffee to improve their mood, depression, and cognitive function; these psychological changes were increased during the quarantine period (57–59); this view can explain the increased consumption of coffee in the adolescent age group in two studies (23, 54).

Three Good quality studies demonstrated a significant decrease in the intake of sugar-added drinks (35, 36, 54). Two other studies reported an increase in sugar-added beverages, but their questionnaire was invalid, and their population was not representative of all the nations' adolescents and children (25, 48).

### Fruits and vegetables

Fresh fruits and vegetables are integral to a healthy lifestyle and diet; they are necessary for a healthy immune system. In this systematic review, twenty studies evaluated the intake of fruits, and eleven studies reported a significant increase in consumption of fruits (22, 23, 25, 26, 28, 37, 46, 48, 49, 51, 54), but four studies reported the opposite change (34–36, 38). Also, five studies did not show significant change during confinements compared to before the pandemic (27, 32, 39, 40, 44). Eighteen studies investigated the change in the consumption of vegetables. Nine articles demonstrated a significant increase (22, 23, 25, 26, 37, 40, 49, 51, 54), three articles showed a decrease (34, 35, 38), and six studies did not report significant change compared to before COVID-19 (27, 32, 39, 44, 48, 55). Finally, we can conclude that consumption of fruits and vegetables increased during the COVID-19 pandemic compared to before the pandemic. During confinement and COVID-19, People tended to change their diet to healthy foods such as fruits and vegetables. Low fruit and vegetable intake in some countries may be explained by lower availability and higher price. In some countries, COVID-19 tremendously affected farms and industries (60). For example, tight quarantine rules in China and the USA led to difficulties in going out of the house, and therefore, the availability of fruits and vegetables decreased (61). Thus, governments have to consider these problems in farming fruits and vegetables and look for solutions for same situation like COVID-19 pandemics may happen in future again.

### Milk and dairy

Findings of studies about dairy and milk intake are disputed. Six studies reported an increase (22, 23, 26, 39, 46, 54). On the other hand, four of them showed a decrease in the consumption of milk and dairy products (28, 32, 34, 35). In some countries, dairy industries were influenced by the COVID-19 pandemic due to the closure of restaurants, worker shortage, difficulties in transportation, etc., (61), so lower intake of dairy products could be explained by this point of view. Generally, we can not find an exact answer to how dairy intake has changed during confinement.

### Protein rich foods

Protein-rich foods play an important role in healthy human nutrition with their high protein, vitamin, and mineral contents. So, protein-rich foods are recommended for a healthy diet (62). In this systematic review, nine articles investigated intake of meat during COVID-19 (22, 23, 26, 34, 35, 39, 48, 49, 51).four studies reported increased intake (23, 39, 48, 49), and three other articles showed decreased intake (22, 34, 35). Also, two articles did not report a significant change in meat consumption during COVID-19 compared to before COVID-19 (26, 51). World disasters and pandemics throughout history have influenced the consumption of protein-rich foods. Therefore, During the COVID-19 pandemic confinements, people were confronted with difficulties. For example, meat producers have faced problems in meat harvesting and shipment of products, and all of these reasons adversely impacted meat production (63). Socioeconomic status (SES) is another factor that plays a crucial role in eating habits. People with disadvantaged SES were more prone to worsen their diet and eating habits (39). Due to the pandemic and restrictions, changes occurred in SES in most families, and pre-existing inequalities were exacerbated (45).

Additionally, included studies showed ambiguous results in consuming egg, fish, and chicken intake. Four studies investigated egg consumption in children and adolescents; three reported an increase, and one reported decreased intake of eggs (22, 23, 34, 54). Seven studies evaluated fish consumption in children and adolescents (22, 23, 26, 28, 34, 39, 54). Three articles showed increased fish intake. Two studies reported a decrease, and two did not report any significant change in fish intake. Only four studies evaluated chicken and poultry intake (23, 26, 35, 39). One study reported an increase, one showed a decrease, and one did not report a significant change. In conclusion, included studies showed various results among children and adolescents. We need further research with higher validity to improve our knowledge about protein-rich food intake change during COVID-19 compared to before the pandemic.

### Legumes and cereals

Six studies reported changes in the consumption of legumes. Children and adolescents significantly increased their consumption of legumes except for Spain and Poland children and adolescents (22, 28, 39, 43, 49, 51). Also, four studies reported changes in bread and bakery consumption during COVID-19 pandemic confinements, and all of them except a study performed in Poland reported a significant increase in consumption of bread and bakery compared to before the pandemic (5, 23, 39, 49). The study was conducted in Poland, used a validated questionnaire, and had a good quality assessment (39); however, the rest of the studies reported that increased bread and bakery consumption have poor qualities (5, 23, 49). Available data for consumption of rice, soybean, and cereal were limited. Just one article investigated soybean intake and reported a significant decrease in consumption before the pandemic (35). Also, just three studies evaluated rice consumption; one reported increased consumption (23), and two studies reported decreased consumption (35, 54). Due to limited data about these food types, we need further research investigating the intake of rice and soybean.

It has been shown that social well-functioning programs and food distribution systems can reduce the adverse effects of food insecurity induced by a different crisis. For example, Productive Safety Net Programme (PSNP) in Ethiopia during the pandemic showed a low risk of food insecurity (9.3%) in participants (64).

### Strengths and limitations

It is important to notice that this systematic review has some strengths and limitations. First, This is the first systematic review that summarizes the documents evaluating the Impact COVID-19 pandemic on children and adolescents' eating habits. Also, this study included many studies from all over the world. However, there were several limitations to this study. Firstly, most of the included studies are cross-sectional, and we recommend performing more longitudinal studies with longer follow up to evaluate longer consequences of dietary change during this period. Secondly, included studies had various designs and methodological aspects, which can make it difficult to reach exact conclusions about dietary changes; thirdly, most of the studies used online surveys for evaluating dietary change due to limitations of lockdowns, which can lead to recall bias in results because there were not any valid type of tool for dietary change assess. Recall bias decreases the value of reported changes because participants are likely to over or underestimate the answers in questionnaires. Included studies were conducted in several countries on the five continents with different cultures and specific eating habits which could be led to some inconsistency among observations.

Additionally, the availability of the internet is limited in lower-income countries and several groups of people. In most of the studies, dietary changes were evaluated in high and middle-income countries. In contrast, there is a greater probability of nutritional changes in low-income countries, especially during quarantine. For example, studies on adult populations in Nigeria, Mexico, and Bangladesh reported a significant worsening of food security status pre and post-pandemic periods based on earlier available data (65–67). The same results were reported for Uganda and Kenya (68).

## Conclusion

The Impact of COVID-19 on children and adolescents' eating habits was both positive and negative, for example, a decrease in fast food, fruits, and vegetable consumption vs. an increase in snacking and sweet consumption. Both changes have significant short-term and long-term impacts on population health. This study could give us clues about changes in eating habits in children and adolescents during confinements. It is worth noting that eating habits established during the pandemic in children and adolescents could affect eating habits in future years and these changes could last until adulthood (20). Thus, we can use them to improve the negative changes during the COVID-19 era.

## Author contributions

MQ and H-SE came with the idea of this article. FP, ME, and AE did the study search and evaluate the articles. FP and ME wrote the manuscript and the tables. MQ and H-SE did the final proof of the article. All authors contributed to the article and approved the submitted version.

## Conflict of interest

The authors declare that the research was conducted in the absence of any commercial or financial relationships that could be construed as a potential conflict of interest.

## Publisher's note

All claims expressed in this article are solely those of the authors and do not necessarily represent those of their affiliated organizations, or those of the publisher, the editors and the reviewers. Any product that may be evaluated in this article, or claim that may be made by its manufacturer, is not guaranteed or endorsed by the publisher.

## Abbreviations

COVID-19: Corona Virus Disease 2019
PRISMA: Preferred Reporting Items for Systematic reviews and Meta-Analyses
SES: Socioeconomic Status
PSNP: Productive Safety Net Programme
FP: Farzad Pourghazi
ME: Maysa Eslami.
