# Supplementary material for: Eating habits of children and adolescents during the COVID-19 era: A systematic review
Source: Front Nutr. 2022 Oct 18;9:1004953. doi: 10.3389/fnut.2022.1004953 (PMC9623566; doi:10.3389/fnut.2022.1004953)
Supplement: Supplementary file 1 [file Table_1.docx]

| **Study** | **Selection** | | | |  | **Compatibility** | |  | **Outcome** | | **Total** |
| --- | --- | --- | --- | --- | --- | --- | --- | --- | --- | --- | --- |
|  | **Representativeness of cases** | **Sample size** | **Non-respondents** | **Ascertainment of the exposure** |  | **On age** | **On other risk factors** |  | **Assessment of the outcome** | **Statistical test** |  |
| Adams 2021 |  |  |  |  |  |  |  |  |  |  |  |
| Adams 2020 | 0 | 0 | 0 | 0 | 0 |  | 0 |  | 1 | 1 | 2 |
| Aguilar 2021 | 0 | 0 | 0 | 0 | 0 |  | 0 |  | 1 | 1 | 2 |
| Alamrawy 2021 | 0 | 0 | 0 | 0 | 0 |  | 0 |  | 1 | 0 | 1 |
| Al Hourani 2021 | 0 | 1 | 0 | 0 | 0 |  | 0 |  | 2 | 1 | 4 |
| Allabadi 2020 | 1 | 0 | 0 | 0 | 0 |  | 0 |  | 0 | 1 | 2 |
| Androutsos 2021 | 0 | 0 | 0 | 0 | 0 |  | 0 |  | 0 | 1 | 1 |
| Beck | 0 | 0 | 0 | 0 | 0 |  | 0 |  | 0 | 1 | 1 |
| Burkart | 0 | 0 | 1 | 1 | 0 |  | 0 |  | 2 | 1 | 5 |
| Cipolla | 0 | 0 | 0 | 0 | 0 |  | 0 |  | 0 | 1 | 1 |
| Dragon 2020 | 0 | 0 | 0 | 0 | 0 |  | 0 |  | 0 | 1 | 1 |
| Ferrante | 1 | 1 | 0 | 0 | 0 |  | 0 |  | 0 | 1 | 2 |
| Giannini | 0 | 0 | 0 | 0 | 0 |  | 0 |  | 0 | 1 | 1 |
| Glabska 2020 | 1 | 1 | 1 | 0 | 0 |  | 0 |  | 2 | 1 | 5 |
| Hanabazaza | 1 | 1 | 0 | 0 | 0 |  | 0 |  | 0 | 1 | 3 |
| Hashem | 1 | 0 | 0 | 0 | 0 |  | 0 |  | 0 | 1 | 2 |
| Horikawa | 1 | 1 | 1 | 0 | 1 |  | 1 |  | 0 | 1 | 6 |
| Jia | 1 | 1 | 0 | 0 | 0 |  | 0 |  | 2 | 1 | 5 |
| Kim | 1 | 1 | 1 | 0 | 1 |  | 1 |  | 2 | 1 | 8 |
| Kolota | 1 | 1 | 1 | 0 | 0 |  | 0 |  | 2 | 1 | 6 |
| Lopez Bueno 2020 | 1 | 0 | 1 | 0 | 0 |  | 0 |  | 0 | 1 | 3 |
| Luszczki | 1 | 1 | 1 | 2 | 0 |  | 0 |  | 2 | 1 | 8 |
| Malta | 1 | 1 | 1 | 0 | 0 |  | 0 |  | 0 | 1 | 4 |
| Maximova | 1 | 0 | 1 | 0 | 0 |  | 1 |  | 2 | 1 | 6 |
| Mazzolani | 0 | 0 | 1 | 0 | 0 |  | 0 |  | 0 | 1 | 2 |
| Medrano | 0 | 0 | 1 | 0 | 0 |  | 0 |  | 0 | 1 | 2 |
| Philippe 2020 | 1 | 0 | 0 | 0 |  | 1 | 1 |  | 2 | 1 | 7 |
| Philippe 2021 | 1 | 0 | 0 | 0 |  | 0 | 0 |  | 0 | 1 | 2 |
| Pietrobelli 2020 | 0 | 0 | 0 | 0 |  | 0 | 0 |  | 0 | 1 | 1 |
| Pujia | 1 | 0 | 0 | 0 |  | 0 | 0 |  | 2 | 1 | 4 |
| Radwan | 0 | 1 | 0 | 0 |  | 0 | 0 |  | 0 | 1 | 2 |
| Ramos Alvarez | 0 | 0 | 0 | 2 |  | 0 | 0 |  | 2 | 1 | 5 |
| Ruiz Rosso | 1 | 1 | 1 | 0 |  | 0 | 0 |  | 2 | 1 | 6 |
| Vall Roque | 1 | 1 | 1 | 0 |  | 0 | 0 |  | 2 | 1 | 6 |
| Welling | 0 | 0 | 0 | 0 |  | 0 | 0 |  | 2 | 1 | 3 |
| Yu | 1 | 1 | 1 | 0 |  | 0 | 0 |  | 2 | 1 | 6 |
| Zachurzok | 0 | 0 | 0 | 0 |  | 0 | 0 |  | 0 | 1 | 1 |

**Table S1.** Newcastle-Ottawa assessment scale for cross-sectional studies.
